# Impact of increasing the availability of healthier vs. less-healthy food on food selection: a randomised laboratory experiment

**DOI:** 10.1186/s12889-020-10046-3

**Published:** 2021-02-01

**Authors:** Rachel Pechey, Olivia Sexton, Saphsa Codling, Theresa M. Marteau

**Affiliations:** 1 Behaviour and Health Research Unit, Institute of Public Health, University of Cambridge, Cambridge, UK; 2 Nuffield Department of Primary Care Health Sciences, University of Oxford, Oxford, UK

**Keywords:** Absolute-and-relative availability, Food selection, Socioeconomic position, Health inequalities, Response inhibition, Food appeal

## Abstract

**Background:**

Environmental cues shape behaviour, but few studies compare the impact of targeting healthier vs. less-healthy cues. One online study suggested greater impact on selection from increasing the number of less-healthy (vs. healthier) snacks. The current study aimed to: (1) extend the previous study by using physically-present snacks for immediate consumption; (2) explore responsiveness by socio-economic position; (3) investigate possible mediators (response inhibition, food appeal) of any socio-economic differences in selection.

**Methods:**

In a between-subjects laboratory experiment UK adults (*n* = 417) were randomised according to their ID number (without blinding) to one of three ranges of options: Two healthier, two less-healthy [“Equal”] (*n* = 136); Six healthier, two less-healthy [“Increased Healthier”] (*n* = 143); Two healthier, six less-healthy [“Increased Less-Healthy”] (*n* = 138). Participants completed measures of response inhibition and food appeal, and selected a snack for immediate consumption from their allocated range. The primary outcome was selection of a healthier (over less-healthy) snack.

**Results:**

The odds of selecting a less-healthy snack were 2.9 times higher (95%CIs:1.7,5.1) in the Increased Less-Healthy condition compared to the Equal condition. The odds of selecting a healthier snack were 2.5 times higher (95%CIs:1.5,4.1) in the Increased Healthier (vs. Equal) condition. There was no significant difference in the size of these effects (− 0.2; 95%CIs:-1.1,0.7). Findings were inconclusive with regard to interactions by education, but the direction of effects was consistent with potentially larger impact of the Increased Healthier condition on selection for higher-educated participants, and potentially larger impact of the Increased Less-Healthy condition for less-educated participants.

**Conclusions:**

A greater impact from increasing the number of less-healthy (over healthier) foods was not replicated when selecting snacks for immediate consumption: both increased selections of the targeted foods with no evidence of a difference in effectiveness. The observed pattern of results suggested possible differential impact by education, albeit not statistically significant. If replicated in larger studies, this could suggest that removing less-healthy options has the potential to reduce health inequalities due to unhealthier diets. Conversely, adding healthier options could have the potential to increase these inequalities.

**Trial registration:**

ISRCTN: ISRCTN34626166; 11/06/2018; Retrospectively registered.

## Background

Non-communicable diseases (NCDs), including diabetes, cardiovascular disease and cancer, cause the majority of premature preventable deaths worldwide [1, 2]. Key determinants of NCDs are behavioural risk factors, including excessive energy intake. Moreover, there are substantial socioeconomic inequalities in these patterns of unhealthy behaviour [3–6], and as a result, in the prevalence of NCDs.

One approach to targeting these behavioural risk factors is to alter the physical micro-environment [7–9], including the availability of healthier vs. less-healthy foods. A recent Cochrane review on the impact of altering availability suggests that such interventions can reduce selection and consumption of targeted food products – although conclusions were limited by the quality and quantity of the included studies, resulting in low overall certainty [10]. There is limited evidence to suggest whether altering the availability of healthier or the availability of less-healthy foods might be more effective. This has important implications for determining the most effective policy strategies to encourage healthier diets. One recent study indicated that people may be more responsive to the number of less-healthy foods available, compared to the number of healthier foods available [11]. In this study, the odds of choosing a healthier option were twice as high with four additional healthier options (vs. two healthier and two less healthy options), while the odds of choosing a less healthy option were four times higher with four additional less healthy options. However, this study was conducted online, with participants not receiving their chosen snack, so it remains unclear whether these results would be found when selecting a physically-present food option.

A possible factor that may contribute to whether people respond differentially to healthier vs less-healthy foods is response inhibition, a core element of executive function that includes being able to resist impulsive behaviour [12]. Response inhibition predicts obesity and food-related behaviour [13–16], but may have a more limited (if any) impact on consumption of healthier foods [17–19]. In addition, the effects of response inhibition may also be moderated by food appeal (defined by Vainik et al. [13] as “how participants value a particular food in comparison to other food items or to non-food alternatives”; one key dimension of which is people’s relative preference for different foods). Those with strong motivation towards less-healthy foods and lower response inhibition may be more likely to make less-healthy food selections and to gain the most weight [20–23].

Response inhibition has been associated with socioeconomic status (SES) at birth [24], and poverty in one’s early years [25]. Food appeal has also been associated with socioeconomic status, with some healthier foods having higher appeal for less deprived individuals [26], which could mean that increasing the availability of healthier foods may be particularly beneficial for those who are less deprived. Together, this suggests the choice of targeting healthier or less-healthy food cues may have implications for the effectiveness of an intervention across socioeconomic groups. Any differential responsiveness is essential to establish in order to select interventions for implementation that will not inadvertently increase health inequalities.

The current study aimed to build on the previous online study [11], but using physically-present foods being selected for immediate consumption. In secondary analyses, this study also explored: (a) the potential moderation of responses to this cue by socioeconomic status, and (b) the roles of response inhibition and food appeal as potential mediators of any influence of socioeconomic status on food selection.

### Primary research question

I. Is food selection more sensitive to the availability of less-healthy foods than the availability of healthier foods?

### Primary hypothesis

1. Increasing the number of less-healthy food items has a larger effect on food selection than increasing the number of healthier food items

### Secondary research questions

II. To what extent is the impact of (i) healthier and (ii) less-healthy food availability on food selection moderated by socioeconomic status?
III. Does response inhibition and/or food appeal mediate any relationship between socioeconomic status and food selection?

### Secondary hypotheses

2.
  - Participants with lower socioeconomic status are less likely to choose healthier foods after seeing a greater number of healthier food options than those with higher socioeconomic status
  - Participants with higher socioeconomic status are less likely to choose less-healthy foods after seeing a greater number of less-healthy food options than those with lower socioeconomic status
3. Response inhibition and food appeal each partially mediate the impact of socioeconomic status on food selection

## Methods

### Design

A between-subjects design was used, with participants randomised to one of three availability conditions, which varied the numbers of healthier vs. less-healthy snacks offered: 2 healthier & 2 less-healthy (reference); 2 healthier & 6 less-healthy (increased less-healthy); 6 healthier & 2 less-healthy (increased healthier). These availability conditions match those implemented in the previous online study altering availability of healthier vs. less-healthy food [11]. As such, this study similarly examines the impact of absolute-and-relative availability of healthier vs. less-healthy food (i.e. simultaneously alters the overall number of options and the proportion of healthier options [27]).

Prior to study recruitment, each ID number to be used was randomised to an availability condition (with equal allocation) by a statistician using computer-generated randomisation sequences. Randomisation was conducted separately for higher education vs. lower education participant IDs, to ensure equal numbers of participants of each education level were randomised to each availability condition, in order to maximise power for the secondary hypotheses. ID numbers were assigned sequentially by the research team upon recruitment (there was no blinding as to allocation).

Testing took place between May and November 2018. The study was pre-registered on the Open Science Framework (https://osf.io/zn567), and retrospectively registered in the ISRCTN registry (10.1186/ISRCTN34626166). Ethical approval was obtained from the Cambridge Psychology Research Ethics Committee (Pre.2018.025).

### Sample

A sample of 417 UK adults was recruited by a market research panel to attend an individual study session in Cambridge, England. For each condition, we aimed to recruit 50% higher and 50% lower SES participants, as defined by highest educational qualification. To maximise the difference in educational level between groups, higher SES was defined as having degree or higher education; and lower SES up to GCSEs (General Certificate of Secondary Education; obtained in the UK at age 16) or equivalent – this meant that those who fell in the middle (had ‘A’ level qualifications or equivalent) were excluded from participation. Participants were reimbursed by the researchers upon completion of the study (receiving £30–£40 GBP in a mix of cash and Love2Shop vouchers for their participation, in line with similar studies [28, 29] – incentives were raised to £40 towards the end of study recruitment to meet the required number of lower SES participants).

The sample size was determined using G*Power (version 3.1.9.2), for a logistic regression testing the Primary Hypothesis, with power of 0.8 and alpha =0.025 (to allow for multiple testing when there are 3 groups, and therefore 2 tests against the reference group), to detect a similar effect size (odds ratio 2.16) to that found in the previous online research [11], using a binomial predictor variable, with balanced groups. The R-squared accounted for by control variables was taken from the previous online study (0.0023), with a baseline probability of choosing a healthier option taken to be 40% (based on the probability when presented with equal numbers of healthier and less-healthy options in the online study). This gives a sample estimate of 265, for a 2-group comparison (i.e. 133 per group). The sample size per group was taken to be 138, to give a slight over-recruitment (to account for potential issues such as internet connection difficulties, task loading errors or completion errors). For the 3 availability conditions, this gave a total required sample of 414.

The market research company invited participants by emailing members of their existing mailing lists, as well as through flyers and adverts. The total number of potentially eligible participants reached by these methods is unknown. There was an attendance rate of 78% (417/537 timeslots) during the study. No participants who attended withdrew from the study. Due to a higher than average attendance rate in the last days of recruitment, our final sample size was 417 participants.

### Measures

Outcome: Snack selection (healthier or less-healthy).

The primary outcome was participants’ selection of a healthier or less-healthy snack food. Participants selected a snack for immediate consumption, with the range offered differing depending on their assigned availability condition.

#### Selection of snack foods to offer

##### Healthier vs. less-healthy

Healthier and less-healthy food options were defined by kcal per pack:

Healthier snack foods: 100 kcal or less per pack.

Less-healthy snack foods: 200 kcal or more per pack.

These kcal limits were chosen to match those used in the previous online study [11]. While energy content does not reflect the full picture with regard to healthiness, it is a proxy used in research studies to indicate healthiness (e.g. [30, 31]). Additional criteria used to select snack food options are described below.

##### Piloting to select snack food options

A pilot study was conducted online to identify food options to be used in the main study. This pilot involved 100 people (equally distributed across three occupational groups: A&B: Higher and intermediate managerial, administrative and professional occupations; C1&C2: Supervisory, clerical and junior managerial, administrative and professional occupations; D&E: Semi-skilled and unskilled manual occupations). Participants rated pictures of foods in terms of their familiarity (5-point scale, from ‘*Very familiar’ – ‘Not at all familiar’*), perceived serving size (‘*One portion’*, ‘*More than one portion’*, or ‘*Less than one portion’*) and perceived healthiness (using 7-point rating scales from *‘Very healthy’* to ‘*Very unhealthy’*). This sample size gave us 80% power to detect a medium effect size difference in perceived healthiness.

Healthier and less-healthy foods were selected to:

1. Match (i.e. show no significant difference) in familiarity (given people may select novel foods when offered free food);
2. All be perceived to be single servings (to ensure that these are appropriate to consume in one sitting);
3. Show a significant difference in ratings of perceived healthiness (to expand our operationalisation of healthiness beyond energy (kcal), and given perceptions of healthiness do not always match actual healthiness).

Both healthier and less-healthy items included a mix of two savoury and four sweet options, but these were not matched on flavour.

The six healthier options were Kallo Sundried Tomato and Herbs Corn and Rice Snacks (25 g); Special K Red Berry Cereal Bar (21.5 g); Soreen Malt Loaf Bar (30 g), Co-op Jumbo Juicy Raisins (30 g); Tyrrells Poshcorn Sea Salted (17 g); Nākd Banana Bread (30 g). The mean energy density of these products was 3.6 kcal per gram (range: 3.0–4.9).

The six less-healthy options were: Reese’s Snack Mix (56 g); Sainsbury’s Taste the Difference Billionaires Slice (60 g); Kettle Chips Ridged Flame Steak (40 g); Hershey’s Cookies and Crème (43 g); Lindt Lindor Milk Chocolate Orange Bar (38 g); Walkers Max Paprika Crisps (50 g). The mean energy density of these products was 5.3 kcal per gram (range: 5.0–6.1).

#### Socioeconomic status

This was assessed via two indicators: (1) highest educational qualification and (2) total annual household income. Participants were recruited based on educational qualifications, but as part of the study they also self-reported educational qualifications and total annual household income. Education was used as the SES indicator in primary analyses, and then replaced by income in a second set of analyses to see if results were consistent across SES indicators.

Index of Multiple Deprivation scores, derived from participants’ postcodes, were intended to be used as a third indicator, but due to a high level of missing data (215/417 (51.6%) missing), this variable was excluded from analyses.

#### Response inhibition

A systematic review of neurobehavioural correlates of eating behaviour identified executive function and food appeal as having consistent and reliable effects on behaviour [13]. In terms of executive function, response inhibition – and in particular the Stop-signal and Stroop tasks – were identified as having one of the most consistent relationships with BMI and eating behaviour.

##### Stop-signal task (Lappin & Eriksen, 1966)

This task measured how well participants were able to break off from making a response to a signal. Arrows within a circle were presented on screen, pointing either left or right. Participants pressed a key corresponding to the direction of the arrow (with responses required within 500 ms). After training (32 trials), within the three experimental blocks (64 trials each) the circle in which arrows were presented turned red in 25% of trials, and for these trials, participants were instructed not to respond. The stop-signal reaction time (SSRT) was used as a measure of response inhibition. This was estimated using the quantile method, in which all reaction times on correct ‘go’ trials were arranged in ascending order, and the reaction time corresponding to the proportion of failed inhibition was selected. SSRT was this quantile reaction time minus the mean stop-signal delay.

##### Stroop interference task

Participants were required to identify the colour (by clicking on the appropriate colour name) of a word or block of colour presented on screen. Words consisted of colour names. In some trials, words matched their display colour (congruent), whereas in other trials the words and display colour did not match (incongruent). Participants underwent a practice session (20 trials), then three experimental blocks of 60 trials each. Scores were based on the difference in mean response time between congruent and incongruent trials.

#### Food appeal

##### Implicit appeal: implicit association task

The Implicit Association Task (IAT) was used to measure how quickly participants were able to categorise pictures of healthier and less-healthy snack foods when instructed to group these as ‘positive’ vs. ‘negative’. This was identified in the review of neurobehavioural correlates of eating behaviour [13] as the task best capturing implicit food appeal.

Participants were asked to categorise four types of stimuli – positive words, negative words, pictures of healthier snacks, pictures of less-healthy snacks – as either ‘positive’ or ‘negative’. Pictures of snacks included the food options offered in the study. For each block, participants were presented with trials comprised of an image from one set of snack food pictures or a word from either the positive or negative word sets. Participants were randomised as to the order in which they completed blocks in which they were asked to categorise: (1) healthier snack pictures as ‘positive’, (2) healthier snack pictures as ‘negative’, (3) less-healthy snack pictures as ‘positive’, (4) less-healthy snack pictures as ‘negative’. Separate IAT scores were calculated for healthier and less-healthy snack foods.

##### Explicit appeal: ratings of enjoyment

Participants were presented with pictures of snack foods (including those offered in this study), alongside the question “How enjoyable is eating this food?” (e.g. [32]). They rated each snack on a 7-point scale from -3 “Very unenjoyable” to 3 “Very enjoyable”.

##### Procedure

Participants were invited to attend a study session at a room hired in a local Cambridge church. They were told they were taking part in a study investigating the effect of snacking on performance in cognitive tasks. This cover story was considered necessary as informing participants that we were interested in their selection of snack would likely alter their behaviour, invalidating our outcome measure. After giving informed consent, participants were asked to complete measures of response inhibition and food appeal on a laptop. They were also asked to rate how hungry they were (using a 7-point rating scale: Very hungry – Very full). Following this, they were offered an array of snack food to choose from (with the selection comprised of a range of both healthier and less-healthy options, determined by their availability condition). To ensure the snacks offered varied within each availability condition, the food items presented to each participant were randomly selected from the pool of available items (using computer-generated sequences). As such, participants could receive any of the healthier or any of the less-healthy items regardless of their availability condition. Snacks were presented on a tray, arranged so that healthier and less-healthy options were evenly distributed across the tray. Participants were asked to select one snack and consume it immediately. They were told they would need to consume all of their chosen snack – in line with the cover story that the study was examining the effect of snacking on cognitive performance (although in practice if a participant expressed a wish to stop eating, the researcher moved them on to the next part of the study). After participants had selected their snack, they were asked to also select a drink as part of an add-on study (reported elsewhere: [33]). Finally, after eating their snack (and consuming as much of the drink as they wanted to), participants repeated the Stroop task, before completing questions on their demographic variables. Participants were then fully debriefed as to the study aims and received payment.

##### Analysis

A *p*-value of 0.05 was used to test the Primary Hypothesis. For the subsequent analyses – testing secondary hypotheses and using income as an alternative indicator of SES – a p-value of 0.00625 was used, to adjust for multiple comparisons (in the OSF pre-registration, a value of *p* = 0.004 was stated; this was recalculated as analyses investigating IMD scores were not conducted and as a result a reduced number of statistical comparisons were made).

#### Primary hypothesis

(*Increasing the number of less-healthy food items has a larger effect on food selection than increasing the number of healthier food items*):

This hypothesis was tested using the coefficients from a logistic regression predicting selection of a healthier (or less-healthy) food option. A factor variable for availability condition was created, with the 2 healthier & 2 less-healthy options condition used as the reference group. Control variables included socioeconomic status, gender, age and hunger.

In order to be able to compare the effects of (1) increasing healthier options relative to having equal numbers of healthier and less-healthy options [i.e. the coefficient for 6 healthier & 2 less-healthy options] to the effects of (2) increasing less-healthy options relative to having equal numbers of healthier and less-healthy options [i.e. the coefficient for 2 healthier & 6 less-healthy options], we ran the regression twice with the outcome coded in each of the two possible ways (predicting selection of (1) a healthier food option, and (2) a less-healthy food option). This gave two coefficients, both predicting the impact of increasing a particular food type on selection of that food type. Stata’s ‘lincom’ command was used to compare these coefficients for any difference in magnitude.

#### Secondary hypotheses

For hypotheses 2a & 2b (*Participants with lower socioeconomic status are less likely to choose healthier foods after seeing a greater number of healthier food options than those with higher socioeconomic status; Participants with higher socioeconomic status are less likely to choose less-healthy foods after seeing a greater number of less-healthy food options than those with lower socioeconomic status*):

We added in interactions between availability condition and socioeconomic status (separately for each of the two indicators) to the logistic regression model outlined for hypothesis 1.

For hypothesis 3 (*Response inhibition and food appeal both partially mediate the impact of socioeconomic status on food selection*):

If there were socioeconomic differences in healthier snack selection, separate mediation analyses were planned to investigate the extent to which (a) response inhibition variables and (b) food appeal variables mediate any relationship between socioeconomic status (each indicator separately) and food selection.

## Results

Table 1 shows the characteristics of participants allocated to each of the availability conditions. Their mean age was 40 (range 18–82), with 44% identifying as male.

**Table 1:** Demographic characteristics

	Availability Condition			Total (*N* = 417)
	Increased Healthier (*n =* 143)	Increased Less-Healthy (*n =* 138)	Equal Healthier and Less-Healthy (*n =* 136)	
**Age** (M (SD))	40.45 (13.43)	41.07 (14.21)	38.49 (13.83)	40.02 (13.83)
**Gender (% (n))**^a^				
Male	42.7% (61)	42.0% (58)	48.5% (66)	44.4% (185)
Female	57.3% (82)	58.0% (80)	51.5% (70)	55.6% (232)
**Education (% (n))**				
Lower (up to GCSEs)	49.0% (70)	51.4% (71)	50.7% (69)	50.4% (210)
Higher (degree or above)	51.0% (73)	48.6% (67)	49.3% (67)	49.6% (207)
**Annual household income (% (n))**				
Up to £17,499	18.9% (27)	18.8% (26)	16.2% (22)	18.0% (75)
£17,500–£29,999	22.4% (32)	18.8% (26)	23.5% (32)	21.6% (90)
£30,000–£49,999	24.5% (35)	27.5% (38)	29.4% (40)	27.1% (113)
£50,000 or higher	26.6% (38)	26.1% (36)	20.6% (28)	24.5% (102)
Don’t know/ Prefer not to say	7.7% (11)	8.7% (12)	10.3% (14)	8.9% (37)
**Ethnicity (% (n))**				
White	84.6% (121)	84.8% (117)	88.2% (120)	85.9% (358)
Mixed Ethnicity	2.8% (4)	2.9% (4)	1.5% (2)	2.4% (10)
Asian	8.4% (12)	8.7% (12)	5.1% (7)	7.4% (31)
Black	2.1% (3)	1.4% (2)	2.2% (3)	1.9% (8)
Other	2.1% (3)	2.2% (3)	2.9% (4)	2.4% (10)
**Hunger** (M (SD))^b^	0.80 (1.06)	0.95 (0.95)	0.72 (1.16)	0.82 (1.06)
**Response inhibition** (M (SD))				
Stroop score^c^	− 277.5 (197.7)	− 295.6 (250.6)	− 287.5 (275.1)	− 286.8 (242.1)
Stop-signal reaction time^d^	257.7 (202.7)	247.7 (157.1)	227.1 (141.5)	244.5 (170.6)
**Explicit food appeal: Enjoyment ratings**^e^ (M (SD))				
Healthier food options	0.71 (0.95)	0.54 (0.82)	0.66 (0.92)	0.64 (0.90)
Less-healthy food options	0.99 (0.99)	1.02 (1.04)	1.12 (0.90)	1.04 (0.98)
**Implicit food appeal: IAT scores**^f^ (M (SD))				
Healthier food	0.21 (0.48)	0.28 (0.48)	0.23 (0.58)	0.24 (0.51)
Less-healthy food	0.18 (0.51)	0.17 (0.55)	0.23 (0.50)	0.19 (0.52)

^a^ No participants selected ‘Other’ as their gender

^b^ 7-point rating scale: 0 was labelled “Neither hungry nor full”; 1 was labelled “A little hungry”

^c^ Mean response time in milliseconds for correct control trials minus mean response time in milliseconds for correct incongruent trials

^d^ Estimated using the quantile method, in which all reaction times on correct ‘go’ trials are arranged in ascending order, and the reaction time corresponding to the proportion of failed inhibition is selected. The stop-signal reaction time is this quantile reaction time minus the mean stop-signal delay

^e^ Mean rating across all six healthier (less-healthy) items (ratings from −3 “Very unenjoyable” to 3 “Very enjoyable”)

^f^ Implicit Association Task scores ((Mean reaction time for the negative pairings minus Mean reaction time for the positive pairings)/Pooled S.D.); positive scores represent positive implicit attitudes towards the food items

### Impact of availability

Overall, 43.9% (*n* = 183/417) of snacks selected were healthier:

- 21.0% in the increased less-healthy options condition;
- 44.1% in the equal healthier and less-healthy options conditions;
- 65.7% in the increased healthier options condition.

Table 2 shows the results of logistic regressions run as part of the primary analysis – predicting healthier snack selection from availability condition and education level.

**Table 2:** Logistic regression predicting healthier option selection (socioeconomic status variable: highest educational qualification)

		Odds Ratio (95% CIs)	***p***-value
Availability condition (*ref: Equal Healthier and Less-Healthy)*	Increased Less-Healthy	0.34 (0.20, 0.60)	< 0.001
	Increased Healthier	2.49 (1.51, 4.10)	< 0.001
Education (*ref: Lower: Up to GCSE)*	Higher: Degree or above	1.10 (0.70, 1.70)	0.688
Age		1.01 (0.99, 1.03)	0.279
Gender (*ref: Female)*	Male	0.57 (0.37, 0.89)	0.013
Hunger^a^		0.80 (0.65, 0.99)	0.036
Intercept		0.78 (0.38, 1.82)	0.572

Pseudo R-squared = 0.1267; Log-likelihood chi-square (degrees of freedom: 6) = 71.17 (*p* < 0.0001); Number of observations = 410 [5 participants did not report age; 2 did not report hunger – 2 of whom were allocated to the Increased Less-Healthy condition, 4 to the Equal Healthier and Less-Healthy condition, and 1 to the Increased Healthier condition]

^a^ 7-point rating scale: 0 was labelled “Neither hungry nor full”; 1 was labelled “A little hungry”

In the increased less-healthy condition, the odds of selecting a healthier option decreased by a factor of 0.34 (or equivalently, the odds of selecting a less-healthy option were almost three times higher (1/0.34 = 2.92; 95%CIs: 1.68, 5.08)), compared to the equal healthier and less-healthy options condition.

In the increased healthier condition, the odds of selecting a healthier option were nearly 2.5 times as high as the odds in the equal healthier and less-healthy options condition.

There were some discrepancies between education group reported by the recruitment agency and self-reported in the questionnaire: 7 participants in the higher education group reported highest qualifications of A-levels or lower (one no qualifications, one GCSEs, five A-levels); 22 participants in the lower education group reported highest qualifications above GCSE-level (18 A-level; 4 degree). Sensitivity analyses removing these participants showed similar results.

Impact of Increasing Healthier Snack Availability vs. Impact of Increasing Less-Healthy Snack Availability [Primary Hypothesis]: Comparing the size of these coefficients suggested no significant difference between the size of the effect of increasing healthier options compared to increasing less-healthy options (difference in magnitude coefficient = − 0.160; 95% CIs: − 1.06, 0.74; *p* = 0.727).

Analysis using income rather than education as the socioeconomic indicator showed similar results (see Supplementary Materials pp.1–3: Analysis by Income).

### Interactions with education [hypotheses 2a and 2b]

In the equal healthier and less-healthy options condition, a higher proportion of the lower education group (54%) selected a healthier option than those in the higher education group (34%). The pattern of healthier option selection was as predicted in the increased less-healthy (14% lower educated and 28% higher educated) and increased healthier (61% lower educated and 70% higher educated) conditions.

**Fig. 1:**
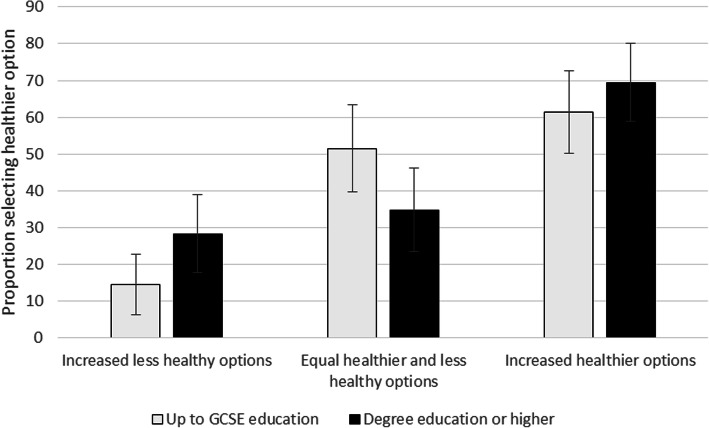
Adjusted proportions of participants selecting healthier options, by availability condition and education (error bars represent 95% CIs)

Figure 1 shows these proportions adjusted for other model covariates – and that the error bars around these estimates were quite wide. The interaction between higher education and increased less-healthy items (Odds ratio: 4.0; 95%CIs: 1.3, 12.4; other model coefficients in Supplementary Materials Table S3) suggested that the impact of this availability manipulation on item selection might be smaller for higher educated participants – as they were more likely to still select a healthier option, but this difference did not reach significance at the *p* = 0.00625 threshold used for these analyses.

The interaction terms also suggested that participants with higher educational qualifications might be more likely to select healthier items in the increased healthier items condition, although this was again not statistically significant (Odds ratio: 2.5; 95%CIs: 0.9, 6.8).

Including the interaction terms in the model meant that the odds ratio for the increased healthier condition – now representing the change for lower educated participants – was reduced and was no longer statistically significant (Odds ratio: 1.6; 95% CIs: 0.8, 3.2). This reflects that most of the change observed between this condition and the reference equal healthier and less-healthy options condition occurred in the higher education group. In contrast, the odds ratio for the increased less-healthy condition increased in size (Odds ratio of 0.17 (95%CIs: 0.07, 0.38) – equivalent to an increase by a factor of 5.9 in the odds of selecting a less-healthy option – compared to around 0.34 in the model without interactions), reflecting the larger change in the lower education group compared to the equal healthier and less-healthy options condition. Analyses using income rather than education again showed similar results (see Supplementary Materials, Table S2).

### The roles of response inhibition and food appeal [hypothesis 3]

We did not observe any socioeconomic differences in selection of healthier options (Tables 2 and S1). As such, no formal mediation analyses were conducted.

Instead, we conducted some exploratory analyses examining the roles of response inhibition and food appeal in food selection in this study:

- Firstly, we explored whether there were any differences between scores on any response inhibition or food appeal variables for those with higher education vs. those with lower education.
- Secondly, we investigated (a) response inhibition and (b) food appeal variables as predictors of healthier food selection, by adding each of these sets of variables separately to the model used for the primary analysis (reported in Table 2).

#### Differences in response inhibition and food appeal by education level

Table 3 shows the results of tests examining whether scores on each response inhibition and food appeal variable differ for those with higher vs. lower education. For response inhibition, these findings suggest no difference in stop-signal response time, but that those with higher education had higher scores (i.e. lower interference effect) on the Stroop task than those with lower education (although this did not reach statistical significance at *p* < 0.00625).

**Table 3:** Response inhibition and food appeal by education level

		Mean (S.D.) *(n)*		Test for difference in means	
		Lower education	Higher education	Test statistic^a^	*p*-value
**Response inhibition**					
Stop-signal response time		243.2 (176.4) *(210)*	245.7 (164.7) *(204)*	χ^2^(1) = 0.457	0.499
Stroop score		− 324.7 (287.4) *(207)*	− 248.3 (177.7) *(204)*	χ^2^(1) = 6.113	0.013
**Food appeal**					
Explicit: enjoyment ratings	Healthier food	0.56 (0.98) *(209)*	0.72 (0.80) *(206)*	χ^2^(1) = 2.021	0.155
	Less-healthy food	1.01 (1.02) *(210)*	1.07 (0.93) *(205)*	χ^2^(1) = 0.344	0.558
Implicit: IAT scores	Healthier food	0.29 (0.51) *(202)*	0.19 (0.51) *(198)*	t(398) = 1.945	0.053
	Less-healthy food	0.29 (0.53) *(193)*	0.09 (0.50) *(185)*	t(376) = 3.708	< 0.001

^a^ Choice of t-test or Kruskal-Wallis determined by normality of response inhibition or food appeal variable, as assessed by Shapiro-Wilk tests and examination of diagnostic plots

In terms of food appeal, group means suggested that those with higher education reported higher explicit food appeal for healthier food than those with lower education, but this difference was not statistically significant. No difference was observed for less-healthy foods. Conversely, for implicit food appeal, no significant difference was observed for healthier foods, while those with higher education had lower IAT scores (i.e. lower implicit appeal) for less-healthy foods compared to those with lower education (t(376) = 3.708; *p* = 0.0002).

#### Response inhibition and healthier food selection

Adding stop-signal response time and Stroop scores to the model for the primary analysis did not alter the coefficients already presented in Table 2. Neither stop-signal response times (Odds ratio: 0.9997; 95%CIs: 0.9984, 1.0010) nor Stroop scores (Odds ratio: 0.9997; 95%CIs: 0.9987, 1.0006) predicted healthier food selection (see Supplementary Materials Table S4 for all coefficients in this model).

#### Food appeal and healthier food selection

Adding explicit and implicit food appeal variables to the model reported in Table 2 reduced the effect size for the increased healthier condition (Odds ratio: 2.12; 95%CIs: 1.16, 3.86). In addition, the effect of the increased less-healthy condition was larger, with an odds ratio of 0.20 (95%CIs: 0.10, 0.40).

Explicit food appeal scores predicted healthier food selection: for every additional one-point increase in mean enjoyment ratings for healthier foods, the odds of selecting a healthier option were 2.79 times higher (95%CIs: 1.97, 3.95); for every one-point increase in mean enjoyment ratings for less-healthy foods, the odds of selecting a healthier option were three times lower (Odds ratio: 0.32; 95%CIs: 0.23, 0.44).

In contrast, implicit food appeal scores did not significantly predict healthier food selection (IAT score for healthier foods: Odds ratio: 0.66; 95%CIs: 0.40, 1.11; IAT score for less-healthy foods: Odds ratio: 0.78; 95%CIs: 0.47, 1.29). (See Supplementary Materials Table S5 for all coefficients in this model.)

## Discussion

The results of this study suggest that increasing the number of healthier options and increasing the number of less-healthy options both had moderate effects (odds ratios equivalent to 2.5 and 2.9) on healthiness of snack selection, compared to being offered equal numbers of healthier and less-healthy options. These are similar in size to the odds ratios found in a previous online study (odds ratios of 2.0 and 4.3) using the same numbers of healthier and less-healthy options between availability conditions [11]. These equate to standardised mean differences (SMD) of -1.38 and -1.60 for the effects of reducing the number of options on selection, comparable to the estimate in the recent Cochrane review for the impact of reduced availability on item selection (SMD: -1.13, 95% CIs: -1.90 -0.37) [10].

In contrast to the previous study, however, the results from the current study do not support the primary hypothesis that increasing the number of less-healthy food items has a larger effect on food selection than increasing the number of healthier food items. While the direction of effect was consistent with this hypothesis in that odds ratios for the increased less-healthy condition suggested greater change than those for the increased healthier condition, differences in effect size were small and statistically non-significant. Both increasing the number of less-healthy options and increasing the number of healthier options increased the healthiness of snack selected, with no evidence for differential responsiveness between these two cues. The difference between these findings and those of the online study could be due to differences in responding when the food options are physically-present. An alternative explanation might be increased social desirability when selecting a food item with another person (the researcher) present. Indeed, participants were more likely to select healthier options in the current study than in the online study (overall 44% selected a healthier option in this study vs. 35% in the online study). Interestingly, odds ratio sizes became more different – and closer to those obtained in the online study – when food appeal was included in analyses (Increased healthier options: 2.1; Increased less-healthy options: 5.0), albeit still with wide confidence intervals.

This study provides one of the first tests of the idea that environmental cues, such as the availability of healthier and less-healthy foods, might be differentially influential for those with higher vs. lower socioeconomic status. Findings were inconclusive with regard to the hypothesised interactions by SES, although the direction of effects was consistent with the study hypotheses. This study was not powered for these interactions, and future larger scale studies are required to establish if any differences are reliably observed. In particular, these differences are important to establish, given substantial socioeconomic inequalities in these patterns of unhealthy behaviour [3–6]. If we think of the increased less-healthy condition as equivalent to current snack availability in many contexts, if pattern of results observed here was replicated more robustly, this might indicate that removing some of the less-healthy options from our environments could in particular benefit lower socioeconomic status groups. In contrast, introducing additional healthier options might be more beneficial to higher socioeconomic groups, and as a result, could potentially widen health inequalities.

No socioeconomic patterning was observed in the selection of healthier items between socioeconomic groups, as measured by either education or income (similar to findings in the previous online study). While data representing multiple shopping trips aggregated over time suggest socioeconomic differences in purchasing of sweet snacks [4], such differences may not be apparent in a single selection task, as used in the current study. Alternatively, it could be that the context in which these selections were made did not allow for any socioeconomic differences to be readily observed. Differences in implicit food appeal, but not explicit food appeal, were observed by education level. It is possible that we would see differences in healthiness of snack selection if participants were under greater cognitive load, but not in this context when making a deliberative decision – reflecting that explicit, but not implicit, food appeal predicted selection in the current study.

### Strengths and limitations

This study offers a robust test of the impact of altering the number of less-healthy options and altering the equivalent number of healthier options, in order to establish the relative impact of each of these interventions. This is the first study to assess this using physically-present food options for immediate consumption. In addition, the study offers a novel exploration of the relative impact of each of these interventions by socioeconomic status, to explore the potential impact of implementing such interventions for the purpose of reducing health inequalities.

Several limitations should be noted: firstly, the experimenter was present in the room when participants were asked to select their snack, which could have led to social desirability influencing participants’ selections. In addition, we did not assess whether participants guessed the study aim – as such, it is possible that participants were not blind to the study hypotheses. Finally, the study was limited to a small number of pre-packaged snack foods, which represent only part of individuals’ diets. Further studies could explore a wider range of food options.

### Implications for research and policy

Findings suggest that altering availability is a promising strategy to change behaviour and improve diets. This offers further support to policies that set limits on the proportion of less healthy meals or snacks that may be offered, such as those for hospitals in the UK [34]. In contrast to the results of a previous online study, we did not find any evidence that participants were more responsive to increasing the number of less-healthy options rather than increasing the number of healthier options. One possibility is that social desirability may have played a role in the current study, reducing selection of less-healthy options. Conducting further studies that attempt to reduce any social desirability effects while using physically-present food options would be helpful to establish whether this is a contributing factor in these results.

In terms of the possible differential responsiveness to the availability conditions by socioeconomic status, these findings need to be tested in larger studies to establish the reliability of these effects. If replicated, these results could have implications for the types of availability interventions that might be best to implement in order to target behaviour change towards more disadvantaged groups. The pattern of results observed here would suggest altering the availability of less-healthy food options would have the potential to reduce health inequalities due to unhealthier diets, whereas targeting healthier food options might exacerbate existing inequalities. Targeting less-healthy food options may be particularly beneficial when the availability of less-healthy options – such as fast food [35, 36] – is already disproportionately high in more deprived areas. However, targeting less-healthy options may be a less appealing strategy for food retailers, for example, given greater potential backlash from customers, than adding in additional healthier options.

In the current study, participants had no limit on the time taken to make their selection, and this may link with the finding that explicit food appeal predicted selection. However, this is unlikely to reflect the range of contexts in which individuals make selections or purchases of food. To better explore the role of response inhibition and implicit food appeal we might need to conduct studies including contexts where participants are likely to select or purchase foods while their cognitive resources are lowered. If response inhibition or implicit food appeal influence behaviour in such contexts, these might reflect contributory factors in previously observed socioeconomic differences in diet.

## Conclusion

A greater impact from increasing the number of less-healthy (over healthier) foods was not replicated when selecting snacks for immediate consumption: both increased selections of the targeted foods with no evidence of a difference in effectiveness. The observed pattern of results suggested possible differential impact by education, which – if replicated in larger studies – could suggest that removing less-healthy options has the potential to reduce health inequalities due to unhealthier diets. Conversely, adding healthier options would have the potential to increase these inequalities. Further studies could explore whether implicit appeal might play a greater role – and perhaps contribute to socioeconomic differences in diet when in contexts characterised by higher cognitive load.

## Supplementary Information

**Additional file 1.**

## Data Availability

The datasets generated during the current study are available in the Open Science Framework https://osf.io/x6nhv/

## Abbreviations

GCSE: General Certificate of Secondary Education
IAT: Implicit Association Task
IMD: Index of Multiple Deprivation
NCDs: Non-communicable diseases
OSF: Open Science Framework
SES: Socioeconomic status
SMD: Standardised mean differences
SSRT: Stop-signal reaction time
