# Supplementary material for: Impact of increasing the availability of healthier vs. less-healthy food on food selection: a randomised laboratory experiment
Source: BMC Public Health. 2021 Feb 1;21:132. doi: 10.1186/s12889-020-10046-3 (PMC7849186; doi:10.1186/s12889-020-10046-3)
Supplement: Supplementary file 1 — Additional file 1. [file 12889_2020_10046_MOESM1_ESM.docx]

**Impact of increasing the availability of healthier vs. less healthy food on food selection: A laboratory experiment**

**Supplementary Materials**

***Analyses by Income***

Primary analysis

Analysis using income as an indicator of socioeconomic status rather than education showed very similar results to the education analysis, although the odds of selecting a healthier option were increased to 2.8 times higher (95%CIs: 1.7, 4.8) in the increased healthier options condition compared to the equal healthier and less-healthy options condition (see Table S1).

**Table S1.** Logistic regression predicting healthier option selection (socioeconomic status variable: annual household income)

|  | | **Odds Ratio**  **(95% CIs)** | **p-value** |
| --- | --- | --- | --- |
| Availability condition  (*ref:* *Equal Healthier and Less Healthy)* | Increased Less Healthy | 0.34  (0.19, 0.61) | <0.001 |
|  | Increased Healthier | 2.82  (1.67, 4.78) | <0.001 |
| Annual household income  (*ref:* *Up to £17,499)* | £17,500-£29,999 | 1.17  (0.58, 2.35) | 0.667 |
|  | £30,000-£49,999 | 0.93  (0.48, 1.79) | 0.827 |
|  | £50,000+ | 1.18  (0.61, 2.30) | 0.624 |
| Age | | 1.00  (0.99, 1.02) | 0.574 |
| Gender  (*ref:* *Female)* | Male | 0.61  (0.39, 0.97) | 0.038 |
| Hunger | | 0.81  (0.65, 1.00) | 0.048 |
| Intercept | | 0.83  (0.34, 2.06) | 0.692 |

Pseudo R-squared=0.1361; Log-likelihood chi-square (degrees of freedom: 8)= 69.74 (p<0.0001); Number of observations= 374

*Primary Hypothesis*: As before, comparing the size of these odds ratios suggested no difference between the size of the effect of increasing healthier options compared to increasing less-healthy options (coefficient= -0.044; 95% CIs: -0.99, 0.91; p=0.928): both appeared similarly effective at increasing the odds of selecting a healthier option.

Interactions with Income

The patterning of results for income was similar to that for education – with reverse patterning seen in the equal healthier and less-healthy options condition (higher proportions selecting healthier options as income decreased) to that in the increased less-healthy options condition (higher proportions selecting healthier options as income increased). No patterning by income group was seen for the increased healthier options condition.

Figure 2 also shows that the confidence intervals for this analysis were very wide. Reflecting this, no significant interactions were found in the model (Table S2), although the direction again suggested that higher income participants were disproportionately more likely than lower income participants to select healthier options in both the increased healthier and increased less-healthy option conditions compared to the equal healthier and less-healthy options condition. Similar changes to the odds ratios for the increased healthier and increased less-healthy conditions between the model with interactions and the model without interactions (reported in Table S1) were observed as those seen in the education analysis (reflecting where most change occurred by socioeconomic group).

**Figure 2**. Adjusted proportions of participants selecting healthier options, by availability condition and household income (error bars represent 95% CIs)

***
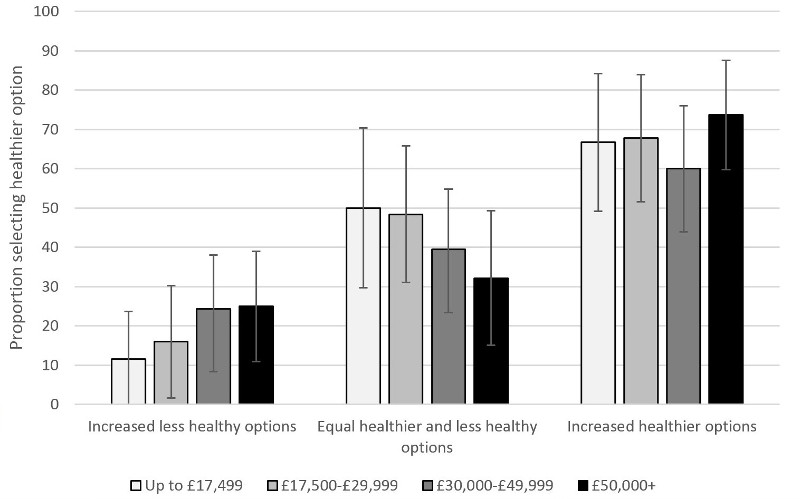
***

**Table S2.** Logistic regression predicting healthier option selection, including interactions between availability condition and annual household income

|  | | **Odds Ratio**  **(95% CIs)** | **p-value** |
| --- | --- | --- | --- |
| Availability condition  (*ref:* *Equal Healthier and Less Healthy)* | Increased Less Healthy | 0.13  (0.03, 0.56) | 0.006 |
|  | Increased Healthier | 2.11  (0.65, 6.91) | 0.216 |
| Annual household income  (*ref:* *Up to £17,499)* | £17,500-£29,999 | 1.00  (0.33, 3.07) | 0.998 |
|  | £30,000-£49,999 | 0.65  (0.22, 1.92) | 0.437 |
|  | £50,000+ | 0.51  (0.16, 1.67) | 0.268 |
| Availability condition by Income interactions | Increased Less Healthy & £17,500-£29,999 | 1.63  (0.23, 11.70) | 0.629 |
|  | Increased Less Healthy & £30,000-£49,999 | 3.66  (0.61, 22.04) | 0.156 |
|  | Increased Less Healthy & £50,000+ | 5.30  (0.83, 33.73) | 0.078 |
|  | Increased Healthier & £17,500-£29,999 | 1.03  (0.21, 5.00) | 0.973 |
|  | Increased Healthier & £30,000-£49,999 | 1.08  (0.23, 4.97) | 0.923 |
|  | Increased Healthier & £50,000+ | 2.76  (0.55, 13.78) | 0.215 |
| Age | | 1.00  (0.99, 1.02) | 0.535 |
| Gender  (*ref:* *Female)* | Male | 0.60  (0.38, 0.96) | 0.033 |
| Hunger | | 0.82  (0.66, 1.02) | 0.077 |
| Intercept | | 1.14  (0.37, 3.55) | 0.818 |

Pseudo R-squared=0.1471; Log-likelihood chi-square (degrees of freedom: 14)= 75.38 (p<0.0001); Number of observations= 374

N.B. This model showed a small increase in terms of the Akaike Information Criterion (AIC), but a substantially higher Bayesian Information Criterion (BIC) to the model without interactions

**Table S3.** Logistic regression predicting healthier option selection, including interactions between availability condition and highest educational qualification

|  | | **Odds Ratio**  **(95% CIs)** | **p-value** |
| --- | --- | --- | --- |
| Availability condition  (*ref:* *Equal Healthier and Less Healthy)* | Increased Less Healthy | 0.17  (0.07, 0.38) | <0.001 |
|  | Increased Healthier | 1.59  (0.79, 3.19) | 0.192 |
| Education  (*ref:* *Lower: Up to GCSE)* | Higher: Degree or above | 0.54  (0.26, 1.12) | 0.098 |
| Availability condition by Education interactions | Increased Less Healthy & Higher Education | 4.04  (1.31, 12.40) | 0.015 |
|  | Increased Healthier & Higher Education | 2.50  (0.91, 6.83) | 0.075 |
| Age | | 1.01  (0.99, 1.02) | 0.324 |
| Gender  (*ref:* *Female)* | Male | 0.60  (0.39, 0.94) | 0.025 |
| Hunger | | 0.80  (0.65, 0.99) | 0.039 |
| Intercept | | 1.11  (0.45, 2.72) | 0.819 |

Pseudo R-squared=0.1385; Log-likelihood chi-square (degrees of freedom: 8)= 77.80 (p<0.0001); Number of observations= 410

N.B. This model showed little difference in terms of the Akaike Information Criterion (AIC), but a higher Bayesian Information Criterion (BIC) to the model without interactions

**Table S4.** Logistic regression predicting healthier option selection, including response inhibition variables

|  | | **Odds Ratio**  **(95% CIs)** | **p-value** |
| --- | --- | --- | --- |
| Availability condition  (*ref:* *Equal Healthier and Less Healthy)* | Increased Less Healthy | 0.32  (0.18, 0.56) | <0.001 |
|  | Increased Healthier | 2.39  (1.45, 3.97) | 0.001 |
| Education  (*ref:* *Lower: Up to GCSE)* | Higher: Degree or above | 1.15  (0.73, 1.80) | 0.543 |
| Age | | 1.01  (0.99, 1.02) | 0.482 |
| Gender  (*ref:* *Female)* | Male | 0.58  (0.37, 0.90) | 0.016 |
| Hunger | | 0.79  (0.64, 0.98) | 0.029 |
| Stop-signal reaction time | | 0.9997  (0.9984, 1.0010) | 0.653 |
| Stroop score | | 0.9997  (0.9987, 1.0006) | 0.512 |
| Intercept | | 0.87  (0.36, 2.10) | 0.753 |

Pseudo R-squared=0.1298; Log-likelihood chi-square (degrees of freedom: 8)= 71.95 (p<0.0001); Number of observations= 405 [Three participants had missing values for SSRT and six for Stroop scores]

N.B. This model had higher Akaike Information Criterion (AIC) and Bayesian Information Criterion (BIC) to the model without response inhibition variables

**Table S5.** Logistic regression predicting healthier option selection, including food appeal variables

|  | | | **Odds Ratio**  **(95% CIs)** | **p-value** |
| --- | --- | --- | --- | --- |
| Availability condition  (*ref:* *Equal Healthier and Less Healthy)* | | Increased Less Healthy | 0.20  (0.10, 0.40) | <0.001 |
|  |  | Increased Healthier | 2.12  (1.16, 3.86) | 0.014 |
| Education  (*ref:* *Lower: Up to GCSE)* | | Higher: Degree or above | 0.86  (0.50, 1.48) | 0.585 |
| Age | | | 0.99  (0.97, 1.01) | 0.398 |
| Gender  (*ref:* *Female)* | | Male | 0.61  (0.36, 1.02) | 0.062 |
| Hunger | | | 0.87  (0.69, 1.12) | 0.283 |
| Explicit: enjoyment ratings | Healthier food | | 2.79  (1.97, 3.95) | <0.001 |
|  | Less healthy food | | 0.32  (0.23, 0.44) | <0.001 |
| Implicit: IAT scores | Healthier food | | 0.66  (0.40, 1.11) | 0.121 |
|  | Less healthy food | | 0.78  (0.47, 1.29) | 0.341 |
| Intercept | | | 4.05  (1.32, 12.38) | 0.014 |

Pseudo R-squared=0.2884; Log-likelihood chi-square (degrees of freedom: 10)= 146.53 (p<0.0001); Number of observations= 370 [Two participants had missing values for both explicit food appeal variables, 17 for healthier IAT score and 39 for less healthy IAT score]

N.B. This model had lower Akaike Information Criterion (AIC) and Bayesian Information Criterion (BIC) to the model without food appeal variables
